# Unexpectedly low UV-sensitivity in a bird, the budgerigar

**DOI:** 10.1098/rsbl.2014.0670

**Published:** 2014-11

**Authors:** Johanna Chavez, Almut Kelber, Misha Vorobyev, Olle Lind

**Affiliations:** Department of Biology, Lund University, SE-221 00 Lund, Sweden

**Keywords:** receptor adaptation, bird senses, UV-vision, colour constancy, spectral sensitivity

## Abstract

Photoreceptor adaptation ensures appropriate visual responses during changing light conditions and contributes to colour constancy. We used behavioural tests to compare UV-sensitivity of budgerigars after adaptation to UV-rich and UV-poor backgrounds. In the latter case, we found lower UV-sensitivity than expected, which could be the result of photon-shot noise corrupting cone signal robustness or nonlinear background adaptation. We suggest that nonlinear adaptation may be necessary for allowing cones to discriminate UV-rich signals, such as bird plumage colours, against UV-poor natural backgrounds.

## 1. Introduction

Photoreceptor adaptation allows vision to cope with differences of several log units in ambient light intensity between night and day and between open and closed habitats [1]. In addition, receptor adaptation contributes to colour constancy, the ability to maintain object colour appearance independent of the illuminating spectrum. This is beneficial because reflectance is invariant under different viewing conditions; a white card appears equally white under a blue sky and in green forest light [2]. Receptor adaptation and colour constancy make colour vision robust and well suited for object classification and identification [3].

Studies of animal colour vision commonly assume that cones adapt to background light (e.g. green vegetation) independently, so-called von Kries adaptation [2]. This leads to a normalization of cone responses and optimal discrimination of stimuli with intensities close to the background. However, the relationship between von Kries adaptation and the complex physiological processes of adaptation [4,5] remains unclear, and experimental tests of von Kries adaptation in humans have produced ambiguous results [6].

Birds have tetrachromatic colour vision mediated by single cones sensitive to ultraviolet (UV), short (S), medium (M) and long (L) wavelengths [7]. We have recently tested spectral sensitivity—the ability for detection of monochromatic stimuli on a grey adaptive background—in budgerigars (*Melopsittacus undulatus*) under different light intensities [8]. At long wavelengths and in bright light (more than 1 cd m^−2^), the results obey Weber's law, i.e. sensitivity—the inverse of detection threshold—is invariantly proportional to background intensity. This is consistent with (i) von Kries adaptation that ensures optimal cone performance during changing light conditions and (ii) invariant signal-to-noise ratio (SNR). By contrast, sensitivity was lower than expected from Weber's law at shorter wavelengths below 450 nm, possibly as a result of photon-shot noise [8]. This noise originates in the stochastic nature of photon arrival at the photoreceptors and is given by the square root of the receptor photon catch, thus affecting vision at low light levels.

Here, we further examine spectral sensitivity at short wavelengths by comparing our preceding results for backgrounds with different intensities but invariant (UV-rich) spectral composition [8], with tests using a bright background without UV illumination. While earlier studies of spectral sensitivity in birds have provided model predictions of how UV-sensitivity may change between UV-rich and UV-poor conditions [9], our study offers the first experimental data on the selective adaptation of UV cones.

## 2. Material and methods

Experiments were carried out using the same experimental set-up, procedures and animals as in an earlier study [8]. For a detailed description of methods see the electronic supplementary material.

### (a) Animals and experimental set-up

We used three male budgerigars kept in a room illuminated by fluorescent tubes set to a 12 L : 12 D cycle. We trained and tested the birds in a cage illuminated from above by light-emitting diodes (LEDs). One cage wall was made from UV-transparent Perspex board (845 mm wide, 652 mm high) covered with white diffusers, and this functioned as adaptive background. The monochromatic stimuli were projected on the left or right side of that background, above two feeders with perches and removable lids. The Swedish Board of Agriculture granted the experiments (M68-11).

**Figure 1.**
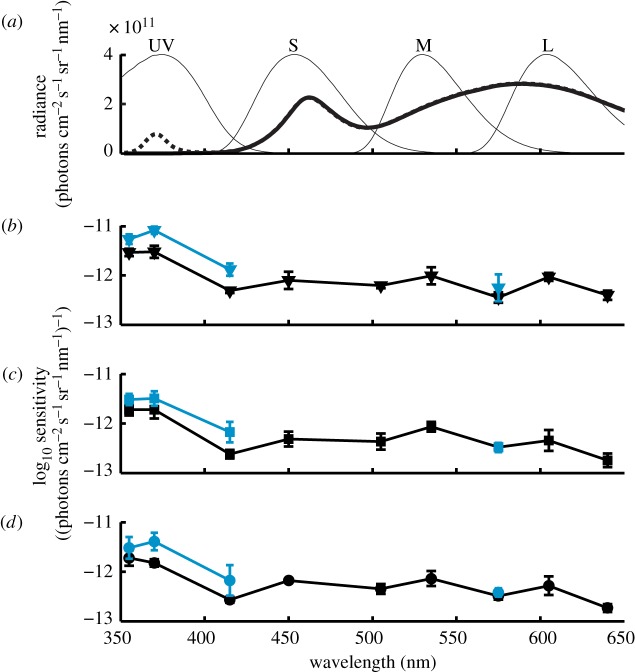
Spectral sensitivity of budgerigars after spectral adaptation. (*a*) UV-rich (dashed line) and UV-poor (solid thick line) illumination, normalized budgerigar cone sensitivities (thin lines). (*b*–*d*) Sensitivity of three birds in UV-rich (black) and UV-poor (grey in print, blue online) condition given as inverse of detection threshold, mean of 4 staircase runs ± s.d. (see the electronic supplementary material for tabulated data). (Online version in colour.)

We used two channels of a 175 W dual power supply (CPX200, Thurlby Thandar instruments Ltd., Huntingdon, England) to control four white LEDs (LZC-00NW40, LED Engin Inc., San Jose, CA, USA) and four UV LEDs (LZ4-00U600, LED Engin Inc.). The white LEDs always generated a luminance of 63.5 cd m^−2^ (4.9 × 10^13^ photons cm^−2^ s^−1^ sr^−1^), while UV LEDs were switched on (1.6 × 10^12^ photons cm^−2^ s^−1^ sr^−1^) or off to create UV-rich and UV-poor test conditions (figure 1*a*).

### (b) Stimuli

Monochromatic stimuli generated by a monochromator (TILL Polychrome V software Polycon v. 3.0 v. 3.0.12, Till Photonics GmbH, Germany) were projected onto the background from behind by two light guides (1000 µm, Ocean Optics). The resulting circular stimuli had approximately Gaussian intensity distributions with a full width at half maximum (FWHM) of 50 mm, they were separated by 280 mm, and 775 mm away from the starting perch. The spectral bandwidth (FWHM) of stimuli was 10 nm except for the stimulus at 415 nm (15 nm FWHM). We measured stimulus (without background illumination) and background radiance (without stimulus) using a spectroradiometer (RSP900-R; International Light, Peabody, MA, USA) aimed at the stimulus centre from 40 mm distance.

### (c) Behavioural procedure

Birds adapted to the cage conditions for 5 min, and each trial was started by an auditory two-tone signal after which the stimulus was presented. Flights from the starting perch to the feeder at the presented light were counted as correct choices and reinforced with 2–4 s access to food. Incorrect choices (flights to feeder with no presented light) were not punished. After each trial, the bird had to return to the starting perch to initiate a new trial. We determined thresholds using a 2-down/1-up staircase procedure with equal step sizes and each staircase comprised 40 trials. We calculated the thresholds as the average intensity of all reversals during the last 20 trials and spectral sensitivity as the inverse of these thresholds.

The spectral sensitivity determined under the UV-rich condition (nine stimuli between 355 and 640 nm) has already been reported [8]. Here, we present new results for subsequent tests during UV-poor conditions, with stimuli at 355, 370, 415 nm, and one control at 575 nm. The birds were first tested for all four wavelengths, and this procedure was repeated until each bird completed four repetitions at each wavelength.

### (d) Receptor adaptation

The intensity difference between background and stimulus equals the intensity of the monochromatic light. In terms of quantum catch, this difference Δ*q_i_* can be expressed as2.1
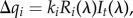
where *R* is the sensitivity of receptor *i* (*i* = UV, S, M, L) and *I_t_* is the intensity of the monochromatic light. Receptor sensitivities were modelled using the Govardovskii template [10] while accounting for oil droplet and ocular media transmittance ([8]; electronic supplementary material). Receptor adaptation *k* is described by von Kries transformation2.2
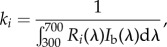
where *I*_b_ is the background intensity. We model spectral sensitivity assuming that detection thresholds are set by receptor noise [11]2.3
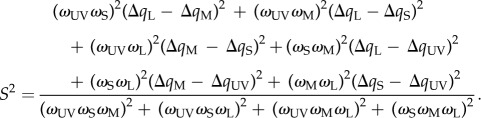


Contrast, *S*, is expressed in the unit of just noticeable difference (JND), where one JND corresponds to threshold. Noise is treated as limiting Weber fractions, *ω*, set to 0.210, 0.121, 0.103 and 0.105 for the UV, S, M and L cones, respectively, as estimated in [8].

## 3. Results

**Figure 2.**
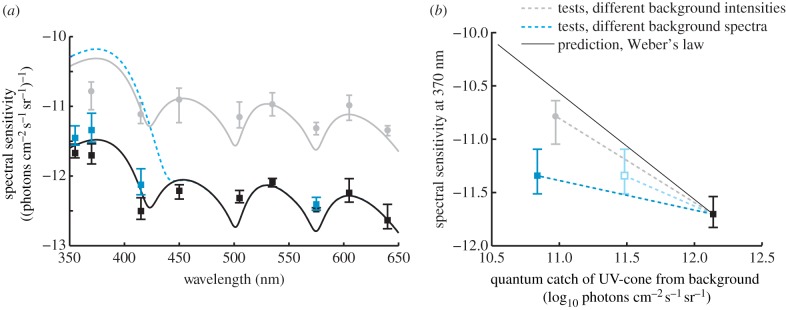
(*a*) Measured average spectral sensitivity of all budgerigars. Lines indicate model predictions and data points indicate measured sensitivity for different backgrounds; black squares: UV-rich bright light; grey squares and dashed line (blue online): UV-poor bright light; grey circles: UV-rich dim light (4.3 cd m^−2^, data from [8]). (*b*) Spectral sensitivity at 370 nm as a function of the quantum catch of the UV-cone. Weber's law predicts a linear relationship with a slope of −1, but we find shallower slopes for the measured data. The light grey open square (light blue online) indicates the relative shift in estimated quantum catch that results from the alternative approach of modelling a 5 nm red-shifted visual pigment using the Lamb-template (see §4). Error bars indicate minimal and maximal individual spectral sensitivity (averages in figure 1). (Online version in colour.)

In tests with UV-poor compared with UV-rich background condition, spectral sensitivity was higher by 0.18–0.44 log units, with averages for all birds of 0.22, 0.36 and 0.38 log units at 355 nm, 370 nm and 415 nm, respectively (figures 1*b*–*d* and 2*a*). We found no differences for the control at 575 nm (figures 1*b*–*d* and 2*a*).

## 4. Discussion

### (a) Unexpectedly low sensitivity in UV-cones

Our results are not consistent with Weber's law, which would predict a linear correlation between background intensity and spectral sensitivity (with a slope of −1 for log units, figure 2*b*). We conclude that either one or other of the assumptions of (i) linear von Kries adaptation or (ii) invariant SNR is erroneous.

We use UV-cone quantum catch to describe background intensity and because the conclusions are similar for all test stimuli (figures 1 and 2), we focus our discussion on the result for 370 nm to simplify the argument. The decrease in quantum catch between the UV-rich and the UV-poor backgrounds was 1.3 log units, but the corresponding average increase in UV-sensitivity at 370 nm was only 0.36 log units and thus lower than expected from Weber's law by 0.94 log units (figure 2*a*,*b*).

In the preceding study of spectral sensitivity in budgerigars for backgrounds of different intensity (but invariant UV-rich spectrum), we found that a 1.17 log unit decrease in UV-cone quantum catch produced an average sensitivity increase of 0.92 log units at 370 nm, thus 0.25 log units less than expected ([8]; figure 2*a*,*b*). We suggested that low UV-sensitivity was the result of photon-shot noise that give lower SNR and signal robustness in dimmer light (lower quantum catch) [8].

Both studies were performed on the same animals following the same experimental methods and similar differences in UV-cone quantum catch between test levels (1.3 versus 1.17 log units). We find no learning effects (see the electronic supplementary material) and no difference in sensitivity at 575 nm (figures 1 and 2), which strongly suggests invariant test conditions. The independence of the behavioural sampling could have been further ensured with interleaved rather than subsequent tests with UV-rich and UV-poor backgrounds, and the possible effects of such methodological differences may be evaluated in future studies. Here, we conclude that the deviation in sensitivity from the Weber's law predictions is larger in the tests with backgrounds differing in UV-composition compared with the tests with different background intensities ([8]; figure 2*a*,*b*).

A possible explanation of our results could be that we have underestimated the UVS cone quantum catch for the UV-poor condition. We used a template suggested by Govardovskii *et al*. [10] to model a visual pigment with peak sensitivity at 371 nm (see §2). It is challenging to estimate pigment sensitivity at short wavelengths below 400 nm, and the template for UV-pigments is less robust than those for pigments sensitive to longer wavelengths [10]. If we instead use a visual pigment template suggested by Lamb [12] and a UV-pigment red-shifted by 5 nm (see the electronic supplementary material for details), UV-cone sensitivity increases at longer wavelengths. This reduces the difference in UV-cone quantum catch between UV-rich and UV-poor conditions to 0.73 log units, which is only 0.37 log units less than expected and close to the deviation measured in the preceding experiments [8]. With these assumptions, all deviations from Weber's law, in both the preceding [8] and present tests, could be explained by a decreasing SNR resulting from photon-shot noise in UV-poor test conditions. Previously, it has been shown that small variation in visual pigment sensitivity has little effect on colour vision modelling under most conditions [13]. The modelling of spectral sensitivity for a UV-poor background is unusual because the predictions change substantially from subtle variation in how pigment sensitivity is estimated (either by Govardovskii or the Lamb-template).

However, if we stay with the initial, and more conventional estimation of UV-cone absorbance, we reach the conclusion that UV-cone adaptation is not consistent with linear independent von Kries adaptation; it is weaker when under spectral changes compared with intensity changes. Could there be any functional advantages of low UV-sensitivity in UV-poor conditions?

### (b) Functional aspects of UV-cone adaptation

At any adaptive state, cones respond over a range of roughly 2 log units of intensity [14,15]. At lower intensities, stimuli are too dim for detection and at higher intensities, cones saturate. Discrimination is optimal at the midpoint of the receptor response function, and linear von Kries adaptation is predicted to keep this midpoint at the intensity of the adaptive background.

Natural backgrounds of green vegetation readily reflect light of wavelengths longer than 400 nm, but barely any UV [16]. The S, M and L-cones adapted to such backgrounds will be able to discriminate most objects, besides extremely bright or dim stimuli (the intensity range of visual scenes sometimes exceeds 3 log units [17]). UV-cones adapted linearly to UV-poor vegetation would optimally discriminate UV-dim objects similar to the background, but saturate for objects that strongly reflect the UV-component of daylight.

Linear and independent von Kries adaptation is in conflict with the observation that many animals communicate with strong UV-signals, such as UV-reflecting feathers in bird mate choice [18] in weakly UV-reflecting vegetation such as the lower strata of the forest. Such communication would require very precise discrimination of signals at intensities far away from the adaptive background.

The nonlinear chromatic adaptation that we may have found in the UV-cones of budgerigars could solve this paradox, keeping the UV-cones in an appropriate state for discriminating UV-rich signals in bright light, despite UV-poor backgrounds. We suggest that this type of discriminative mechanism can only originate from an interaction between cone types during adaptation.

## Supplementary Material

Supplementary methods

## Supplementary Material

Supplementary data
