# Supplementary material for: Unexpectedly low UV-sensitivity in a bird, the budgerigar
Source: Biol Lett. 2014 Nov;10(11):20140670. doi: 10.1098/rsbl.2014.0670 (PMC4261861; doi:10.1098/rsbl.2014.0670)
Supplement: Supplementary methods [file rsbl20140670supp1.pdf]

Supplementary material and methods

Animals

The budgerigars were unrelated male adults housed in separate cages together with female company. Two budgerigars had wild type colouration and one was a blue morph. All birds had experience from earlier psychophysical experiments. Room illumination (Philips Master TL-D 90 Graphica 36W/965, corresponding to a colour temperature of 6500 K) was set to a 12h light/12h dark cycle with 30 minutes transitions mimicking "dawn" and "dusk". The diet was seed mixes together with supplementary vitamins, minerals, fruit, and vegetables.

Experimental set up

The cage was placed in a lightproof compartment in the same room as the housing cages, and the birds could vocally communicate throughout the experiments. The illumination LEDs were aimed upwards so that light reached the cage indirectly from the reflection off wrinkled aluminium foil. This provided homogenous light conditions within the cage. The output of the LEDs change as the equipment get warmer, therefore, we turned on the equipment an hour prior to the tests to ensure stable lighting conditions.

The light from the monochromator was transmitted with a light guide to a system of three aligned UV-transparent lenses that focussed the light upon the entry of a rotating holder (LINOS microbench). This lens system allowed for the insertion of neutral density filters for output intensity control. We varied stimulus intensity with such neutral density filters (LEE) and by adjusting the monochromator output. With the rotating holder, either of two 1000 μm light guides could be placed in the focal plane of the lenses to collect the light and project it upon the adaptive background through aluminium tubes covered with LEE diffusion filters. We covered the aluminium tube exits and the adaptive background with UV-transparent white diffusion filters (a combination of LF416, LF251, and LF252, LEE Filters, Hampshire, UK), which resulted in diffuse stimuli having approximately Gaussian intensity distributions. All light measurements of the adaptive background light and the monochromatic stimuli were captured from an orthogonal position in relation to the surface of the background Perspex board.

Black cloth covered the backside of the background board except for the entry of the stimulus tubes, and we monitored the birds indirectly with a camera placed at the back of the cage.

Behavioural procedure

Each bird was tested in the morning and in the afternoon with a 6 hours break during midday when budgerigars normally are less active. The birds were always allowed to respond to the stimuli presentations without any time-constraint. We did not punish incorrect choices. Instead we removed the stimulus and waited for the bird to return to the starting perch before we initiated a new trial. We adjusted the starting intensities of each staircase so that positive responses were favoured during the first 20 trials while the remaining trials produced about 50% correct choices. The budgerigars were well trained from extensive tests of spectral sensitivity during UV-rich light conditions [8], and there was no need for additional training before the new tests during UV-poor light conditions commenced.

All previous tests reported earlier [8], and the new tests reported here, were carried out in a sequence over a period of about 8 months without pause. To control for any

learning effects, we compared early and late trial but without finding any bias (supplementary data).

Photoreceptor sensitivity

Visual pigment sensitivity is described by the Govardovski template [10] and measured values of visual pigment peak sensitivity of 371 nm, 440 nm, 499 nm and 566 nm for the UV, S, M, and L cones respectively (Bowmaker et al., 1997). Oil droplet transmittance is described by an analytical approximation suggested by Hart and Vorobyev (2005), and values of λ_{cut} and λ_{mid} of 411/431, 497/517, 568/591 for the C, Y and R-type oil droplets respectively [8]. Earlier studies have shown that there is no difference in retinal oil droplets between budgerigar morphs (Knott et al., 2012). The transmittance of the ocular media was taken from (Lind et al., 2014).

As an alternative estimation of UV-cone absorbance, we combine Lambs template [12] to describe the alpha-band of visual pigment absorbance, and the Govardovskii template [10] for beta band absorbance. In reality, beta band absorbance is of little importance due to the filtering of the ocular media, and the major difference between the templates is the narrower alpha-band in the Govardovskii-template because a wavelength dependent parameter used to correct for systematic shape differences of the pigment spectrum at short wavelengths [10]. We also shifted the spectral position of the visual pigment 5 nm towards longer wavelengths in order to increase long-wavelength absorbance. The resulting cone sensitivity is given in figure S1.

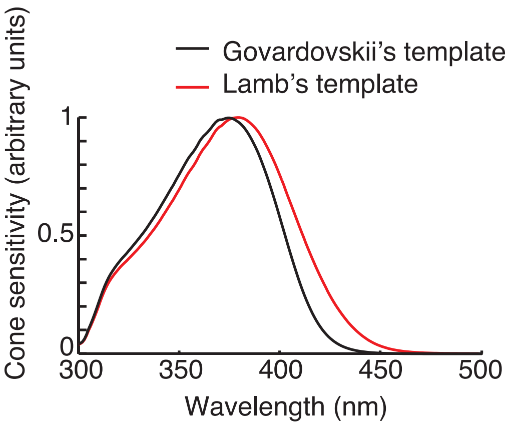

Figure S1. The sensitivity of the UVS cone as estimated by the Govardovskii-template or the Lamb-template together with a 5 nm red-shift. See text for details.

References

Bowmaker JK, Heath LA, Wilkie SE, Hunt DM. 1997. Visual pigments and oil droplets from six classes of photoreceptor in the retinas of birds. *Vision Res.* **37**, 2183-2194.

Hart NS, Vorobyev M. 2005. Modelling oil droplet absorption spectra and spectral sensitivity of bird cone photoreceptors. *J. Comp. Physiol. A* **191**, 381-392.

Knott B, Bowmaker JK, Berg ML, Bennett ATD. (2012) Absorbance of retinal oil droplets of the budgerigar: sex, spatial and plumage morph-related variation. J Comp Physiol A **198**, 43–51.

Lind O, Mitkus M, Olsson P, Kelber A. 2014. Ultraviolet vision in birds: the importance of transparent eye media. *Proc. R. Soc. B* **281**, 20132209.
